# Altered postural sway following fatiguing foot muscle exercises

**DOI:** 10.1371/journal.pone.0189184

**Published:** 2017-12-07

**Authors:** Keiji Koyama, Junichiro Yamauchi

**Affiliations:** 1 Toin University of Yokohama, Kanagawa, Japan; 2 Graduate School of Human Health Science, Tokyo Metropolitan University, Tokyo, Japan; 3 Research Center in Back, Neck, Other Joint Pain and Human Performance (BNOJPH), Khon Kaen University, Khon Kaen, Thailand; West Virginia University, UNITED STATES

## Abstract

This study investigated the acute effects of fatiguing foot muscle exercises on the maximum muscle strength of the foot and postural control ability. Eighteen healthy young individuals performed fatiguing foot muscle strength exercises, and their toe flexor and ankle plantar flexor strength and postural control ability were measured before and after the exercises. Postural control ability was evaluated using the path of the center of pressure (COP) during three balance tasks: double-leg standing with eyes open; double-leg standing with eyes closed; and single-leg standing with eyes open. After the exercises, the muscle strength of both the toe and ankle plantar flexor significantly decreased. Under all of the conditions, most COP variables did not significantly differ before and after the exercises; however, the total length and mean velocity in the single-leg standing with eyes open significantly decreased after the exercises. Postural sway velocities in the anteroposterior direction of double-leg standing with eyes closed and in both anteroposterior and mediolateral directions of single-leg standing with eyes open significantly decreased after the exercises. The associations between relative changes in muscle strength after the exercise and relative changes in COP variables after the exercise were not found. These results indicate that postural control while standing could be maintained even though foot muscle strength is decreased after fatiguing foot muscle exercises.

## Introduction

Foot muscle strength is important for human physical performance involving upright standing and locomotion. Toe flexor muscle strength is an indicator of foot muscle strength, which is a critical factor for enhancing physical performance in adults [1], and it was positively correlated with dynamic lower-limb physical performance, such as sprinting and jumping, in children [2] and adolescents [3]. In the push-off phase of the human gait, when the heel leaves the ground and the dorsiflexion of the metatarsophalangeal joint increases [4], the toe flexors are activated simultaneously [5]. In accordance with these studies, the maximum force generated by the toe flexors at the metatarsophalangeal joint was related to the cross-sectional areas of both the intrinsic and extrinsic muscles of the foot in adults [6]. The toe flexor muscles are an aggregation of muscles that cross the ankle and the metatarsophalangeal joints and are divided into two muscle groups: the intrinsic and extrinsic plantar muscles [6]. The intrinsic plantar muscles are complex multi-segmental structures that have both their origins and insertions within the foot, while the extrinsic plantar muscles have functional roles at the ankle joint. These intrinsic and extrinsic plantar muscles support the unique arch structure of the foot. The muscles of the foot arch and the ankle joint are thought to act like springs and regulate the ground reaction force during standing and movement [7], and active alterations in the muscle length of lower leg are key to maintaining postural balance at the ankle joint while standing [8]. The activities of the intrinsic and extrinsic plantar muscles contribute to postural stability during upright standing, especially in the single-leg standing [9]. Foot muscle strength is considered to be one of the important essentials that provides postural control while standing [10]; however, the relationship between foot muscle strength and postural control remains a subject of discussion.

Physical exercises cause muscle fatigue, which is a decrease in the ability of muscles to produce force [11] and an impairment in the ability of nerves to transmit signals [12]. A loss of foot sensory input is a possible cause of impaired postural balance during upright standing [13]. Additionally, studies have demonstrated that exercises that fatigue the lower leg muscles around the ankle increase the center of pressure (COP) excursion during standing compared with pre-fatigue conditions [14,15]. COP excursion represents the derivation of three components of force or the small slow irregular sway that is generated by the body on the force platform, and it is commonly used as an index of postural stability during standing [16]. Other studies showed either a decrease or no change in COP variables during standing after fatiguing calf-raises [17] and leg cycling [18] exercises. Balance ability can be impaired by dysfunctions of the vestibular, visual, somatosensory, and musculoskeletal systems [19]. Some of these dysfunctions can be explained by impairment of both motor and sensory functions in the foot. Therefore, a change in postural control could be related to decrease in force production by the muscles after they are fatigued. However, there are no studies examining how fatigued muscles of the foot–ankle complex affect postural control during double- and single-leg standing. Knowledge of the influence of fatigued foot muscles on postural control ability will contribute to the understanding of the role of foot muscle strength in postural control and may provide new information that could improve postural balance ability in sports and recreational activities. Therefore, the aim of this study was to investigate the immediate effects of fatiguing foot muscle exercises on foot muscle strength and postural control during standing. It was hypothesized that fatiguing foot muscle exercises would induce a decrease in foot muscle strength and decreased foot muscle strength after the exercises would have cause a change in postural control during standing after the exercises.

## Materials and methods

### Subjects

Eighteen healthy young individuals (age, 21.11 ± 1.45 years; height, 1.69 ± 0.05 m; body mass, 63.86 ± 9.96 kg) were recruited. None of the subjects were taking medications or had peripheral nerve dysfunction, other neurological disorders, or any injuries of the feet or legs within the past year. Subjects were either sedentary or mildly active but were not currently involved in any type of exercise or training program of over 30 minutes per day, 2 days per week. After being deemed eligible, no subjects dropped-out of the study. This study was approved by the Toin University of Yokohama Human Research Ethics Committee in accordance with the Declaration of Helsinki. All subjects gave written informed consent before the onset of the study. Potential volunteers aged between 20 and 25 years old were verbally recruited from a class at the local university during the fall semester (October 2015). The experimental sample size was estimated from previous study data [20] that twenty subjects were measured score of balance ability using balance assessment system before and after the lower extremity fatigue. A significance level of less than 0.05 (Zα / 2 (0.025) = 1.96) and a test power of 80% (Zβ) 0.2 (= 0.84) were used in the calculations, and 16 subjects were added to compensate for the 10% dropout rate since the subjects could have complications during the exercise program. The minimum required sample size was 17 to 18.

### Experimental procedure

**Fig 1:**
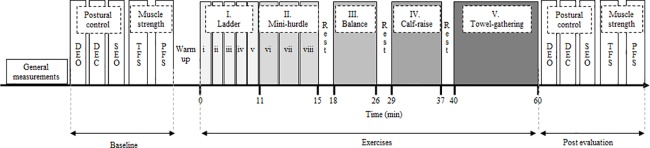
Experimental protocol. DEO, double-leg standing with eyes open; DEC, double-leg standing with eyes closed; SEO, single-leg standing with eyes open; TFS, toe flexor strength; PFS, plantar flexor strength; I. Ladder: i) jogging forward * 4 sets; ii) step running forward * 4 sets; iii) front hopping * 4 sets; iv) cross steps * 4 sets; v) sidesteps * 4 sets; II. Mini-hurdles vi) running forward * 3 sets; vii) front hops * 3 sets; and viii) lateral hops * 3 sets. Ladder drill was repeated 4 times with a 1-minute rest period between different type of drills, and mini-hurdles drill was repeated 3 times with a 1-minute rest period between different type of drills. III. Balance * 3 sets with a 1-minute rest between sets; IV. Calf-raise * 3sets with a 1-minute rest between sets; V. Towel-gathering left and right * 3sets with a 20-second rest between sets for each foot.

Subjects assessed their postural control ability, maximum toe flexor strength (TFS) and maximum ankle plantar flexor strength (PFS) barefoot before and immediately after they performed the exercise program. The exercise program consisted of strengthening exercises for the foot and ankle muscles (Fig 1); the exercise was performed in the following order: 1) agility, 2) balance ball, and 3) foot and ankle muscle strength exercises and the total duration of the whole exercise was 60 min on average. Additionally, our pilot study (n = 12) assessed the cardiovascular fatigue level of the subjects during the exercise and showed that after 1) agility, 2) balance ball, and 3) foot and ankle muscle strength exercises, subjects reached the levels of heart rate; 1) 164.25 ± 30.69, 2) 123.58 ± 20.11, 3) 115.33 ± 23.31 beats/min. and rate of perceived exhaustion (RPE); 1) 16.58 ± 2.57, 2) 13.42 ± 1.88, 3) 12.33 ± 1.92 Borg scale (6–20). Exercise intensities after each exercise were determined by the Karvonen formula and they were on average 1) 71.98 ± 23.36, 2) 38.08 ± 13.41, and 3) 31.68 ± 13.66%.

#### Exercise protocol

Before the exercise, subjects warmed up with self-paced walking for 3 minutes and stretched the foot and leg muscles. The agility exercise consisted of a series of ladder and mini-hurdle drills [21], including: i) jogging forward—ladder; ii) step running forward—ladder; iii) front hopping—ladder; iv) cross steps—ladder; v) zigzag sidesteps—ladder; vi) running forward—mini-hurdles; vii) front hops—mini-hurdles; and viii) lateral hops—mini-hurdles. After subjects performed a few submaximum efforts to familiarize themselves with the drills, they were instructed to perform all of the exercises as quickly as possible. The ladder exercises used a 4.05-m-long ladder with 45-cm-long steps, and the mini-hurdle exercises used 15 mini-hurdles placed at 70-cm intervals. The ladder drill was repeated 4 times with a 1-minute rest period between different type of drills, and the mini-hurdles drill was repeated 3 times with a 1-minute rest period between different type of drills. Next, the balance ball exercise was performed on a “both-sides-up” balance device (BOSU; Fitness Quest, Canton, OH). Subjects were instructed to stand with both feet on the circular BOSU platform and to maintain standing balance over an inflated hemisphere for 2 minutes. This was repeated three times with a 1-minute rest between sets. Last, the standing calf-raises and towel-gathering strength exercises were performed. For the calf-raises exercise, subjects stood with their feet shoulder-width apart and raised their heels with their knees locked. This exercise included three sets of 60 reps per set, with a 1-minute rest between sets. The towel-gathering exercise involved toe flexion at the metatarsophalangeal joint. Subjects pulled a towel that was loaded with a weight equal to 60% of the maximum TFS, with only their toes. This exercise consisted of three sets of 30 reps with a 20-second rest between sets for each foot.

### Measuring maximum isometric TFS and PFS

The maximum voluntary isometric TFS in the standing position was measured with a toe grip dynamometer (T.K.K.3361, Takei Scientific Instruments, Niigata). The details of the apparatus and methodology and the reproducibility of the measurement have been described elsewhere [2,3,6,22]. The range of force with this dynamometer is 1–800 N. The dynamometer consists of strain gauge force transducers, and the force was measured when the grip bar was pulled. The foot was placed on the dynamometer and fixed using the heel stopper with the subject in the standing position with full extension of the knee and hip joints. During the measurements, subjects placed their arms in front of their chests and were instructed to perform the task without extending their hip joints. The opposite foot was positioned beside the dynamometer. The maximum voluntary isometric PFS was measured with a digital force transducer (QTM-05F, Alcare, Tokyo). The range of force with this device is 1–1323 N. To measure PFS, subjects were seated with the hip joints at 90 degrees of flexion and full extension of the knees; they placed their arms in front of their chests. Before measuring both TFS and PFS, subjects performed 3–5 trials at a submaximum isometric force, and then subjects exerted a maximum force on the dynamometer for ~3 s. Both TFS and PFS were measured 5 times with at least a 1-minute rest between bouts. The mean values of the three measurements of maximum force, after excluding the largest and smallest values among the five trials for each foot, were averaged and used for further analysis.

### Evaluation of postural control

Postural control was evaluated using the path of COP. COP was measured on a force plate (9281E, Kistler, Winterthur) under three conditions: double-leg standing with eyes open (DEO); double-leg standing with eyes closed (DEC); and single-leg standing with eyes open (SEO). The order of each condition tested was chosen randomly. The dominant leg was selected for the SEO task. Leg dominance was determined after performing three trials of three functional tests [23]. First, subjects were asked to step onto a 40-cm box; the leg used to perform the step-up was identified as the dominant leg. Next, subjects were pushed from behind, and the leg that stepped out was identified as dominant. Then, subjects were asked to kick a soccer ball, and the leg used to kick the ball was recorded as the dominant leg. The leg that was dominant in two out of the three tests was considered the dominant leg for this study.

To attain the double- and single-leg stance position, subjects were instructed to keep their standing leg still, with their arms by their sides. For single-leg standing, they maintained the non-weight-bearing leg in a position of 90 degrees of knee flexion, keeping their thighs vertical. Before the test measurements were conducted, subjects practiced 3–5 trials of each test position. For the test measurements in each position, subjects were asked to stand for as long as possible, up to 30 seconds. The test was stopped when subjects were unable to maintain the requirements of the test position. The standing test measurements were performed once in each test position with a 10-second rest between each position, which were performed in a random order.

**Fig 2:**
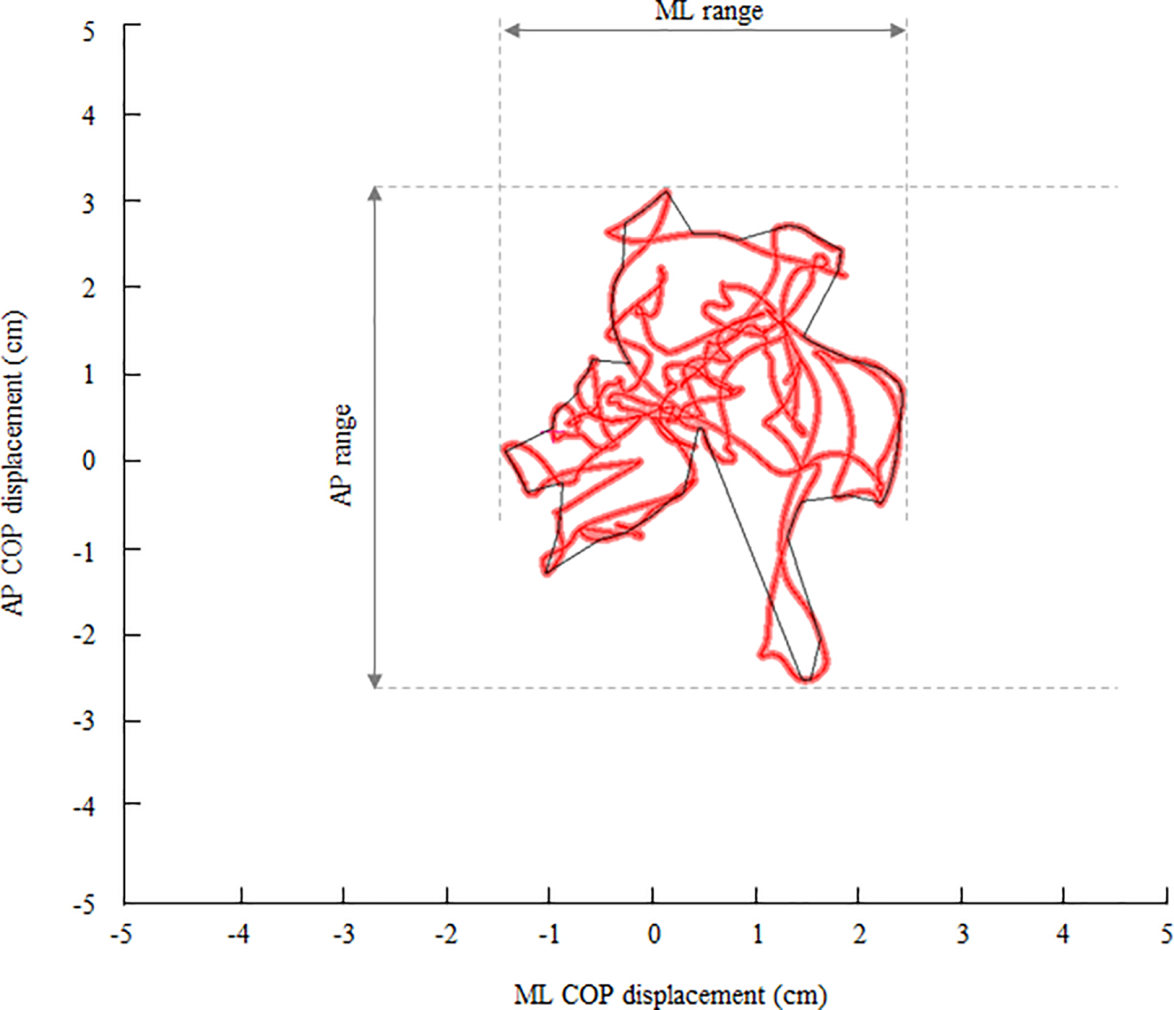
Typical examples of the center of pressure (COP) trajectory during single-leg standing with eyes open task before the exercises. SEO, single-leg standing with eyes open; AP, anteroposterior direction; ML, mediolateral direction; COP trajectory was red line and the total length of the COP trajectory was TL. Black area was sway area of COP (SA). AP and ML ranges were the distance between the anterior and posterior peak displacements and the distance between the medial and lateral peak displacements.

To process the data, the output from the force plate was introduced to the computer through an analog-to-digital converter (PH-770, DKH, Tokyo) at a sampling frequency of 1 kHz., and the COP trajectory was analyzed for 20 seconds, excluding the first 5 and last 5 seconds during double- and single-leg standing tasks. Fig 2 shows typical examples of the COP trajectory before and after the exercises. The following variables were used to describe the movement of the COP that were analyzed by TRIAS software (DKH Corp., Tokyo): total length (TL); mean velocity (MV = TL / total time); sway area (SA); maximum range of anteroposterior sway (AP range); maximum range of mediolateral sway (ML range); mean velocity of anteroposterior sway (AP velocity = AP length / total time); and mean velocity of mediolateral sway (ML velocity = ML length / total time). TL and SA were calculated with the following equation [24]:

$$\mathrm{TL}=\sum_{n=1}^{N-1}\sqrt{{\left(AP_{n+1}-AP_{n}\right)}^{2}+{\left(ML_{n+1}-ML_{n}\right)}^{2}}$$

$$\mathrm{SA}=\frac{1}{2}\sum_{n=1}^{N}\left|AP_{n+1}ML_{n}-AP_{n}ML_{n+1}\right|$$

where N is the number of data points included in the analysis (20000 points) and n is the COP time series. The COP (AP and ML) time series were passed through a Butterworth low-pass digital filter with a 6 Hz cut-off frequency. TL is the total length of the COP trajectory; i.e., the sum of the distance between consecutive points of the COP trajectory. SA estimated by the area of a convex hull; the sum of the triangulation formed by two points on the COP trajectory necessary for calculating the convex hull. AP and ML ranges were the distance between the anterior and posterior peak displacements and the distance between the medial and lateral peak displacements.

### Data analysis

The data are presented as the means and standard deviations (SDs). The significance of differences among parameters between before and after the exercises were tested using a paired t-test. COP variables were determined using two-way analysis of variance [vision (DEO vs. DEC) × exercise (before vs. after)]. The independent variables were vision and exercise, and the dependent variables were COP variables. When an interaction was identified, Bonferroni-corrected pairwise post hoc comparisons were performed. Relationships between relative changes in muscle strength (TFS and PFS) after the exercise and relative changes in COP variables of each standing condition after the exercise were examined using Pearson’s correlations. For SEO, the values of the dominant leg were used for TFS and PFS. The effect size (ES: Cohen’s d) was calculated to examine the average effect of the exercise. Statistical significance was set at p < 0.05.

## Results

Both TFS and PFS significantly decreased after the exercises compared with before the exercises (TFS: n = 18, Pre 212.72 ± 41.81 vs. Post 198.73 ± 45.34 N, p < 0.01, ES = 0.92; PFS: n = 18, Pre 1102.72 ± 178.49 vs. Post 993.59 ± 181.84 N, p < 0.01, ES = 0.75).

**Fig 3:**
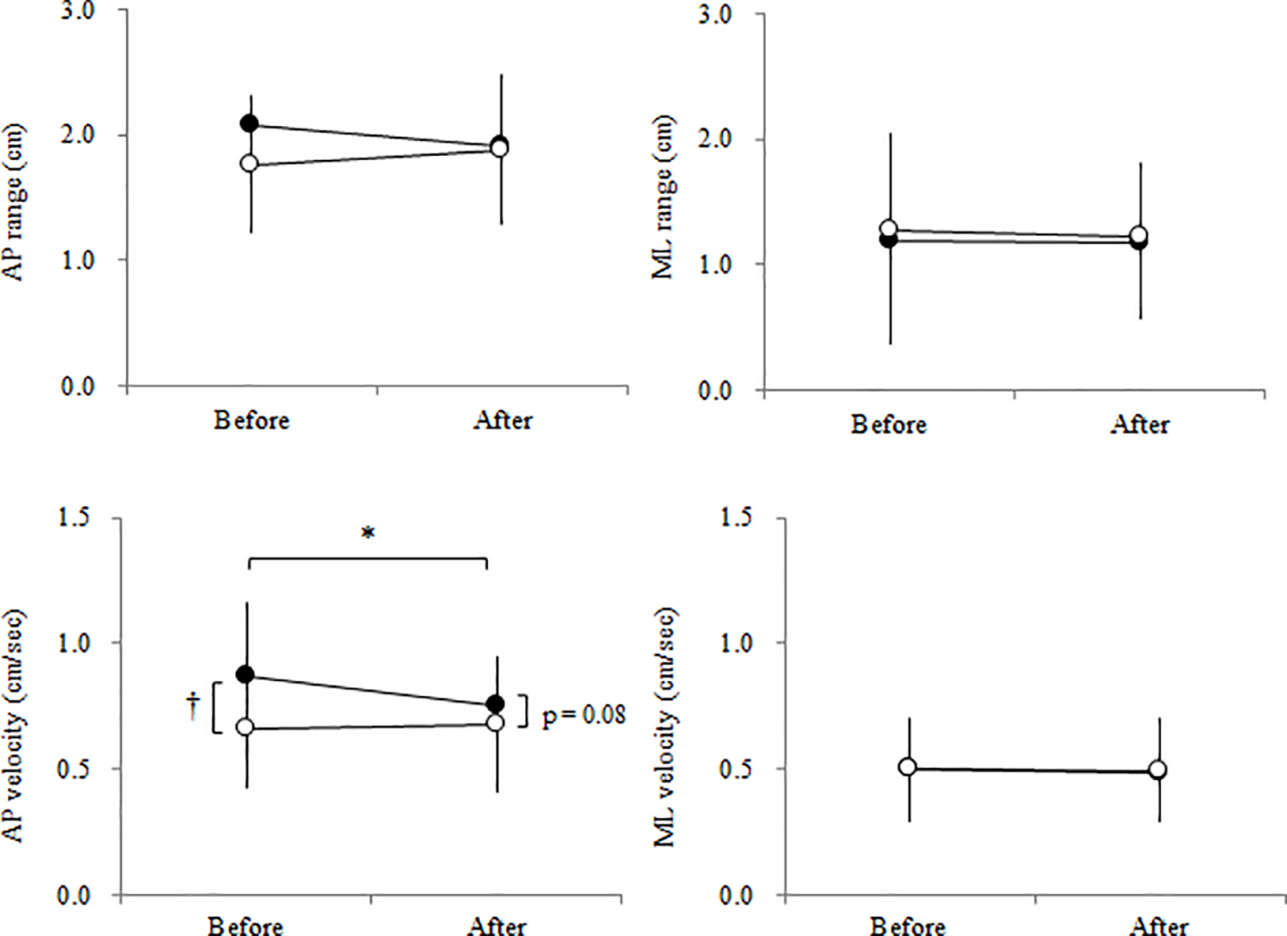
Center of pressure variable in the anteroposterior (AP) and mediolateral (ML) directions before and after exercise in the double-leg standing with eyes open (DEO, ○) and with eyes closed (DEC, ●) conditions. Values are presented as the means and SDs. * denotes a significant difference between before and after at p < 0.01. † denotes a significant difference between DEO and DEC at p < 0.01.

**Fig 4:**
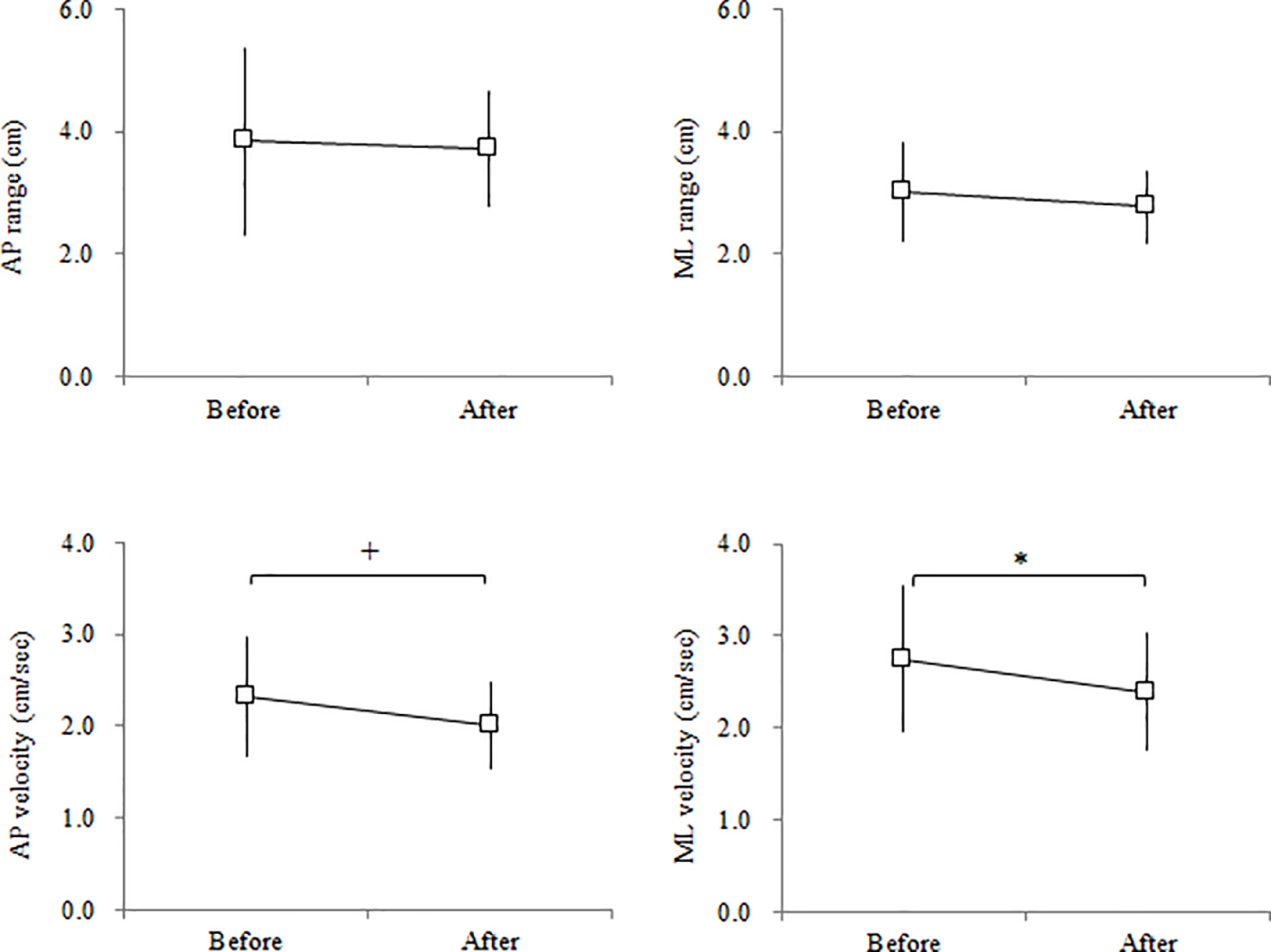
Center of pressure variable in the anteroposterior (AP) and mediolateral (ML) directions before and after the exercise in the single-leg standing with eyes open (SEO) condition. Values are presented as the means and SDs. * and + denote a significant difference between before and after at p < 0.05 and p < 0.01, respectively.

As shown in Table 1, TL and MV in SEO significantly decreased after the exercises compared with before the exercises (n = 17, p < 0.01, ES = 0.94). TL and MV significantly differed between DEC and DEO before the exercises (n = 18, p < 0.01, ES = 0.48), but they did not differ between the two conditions after the exercises. There were significant differences in all of the COP variables between double-leg standing (DEC and DEO) and single-leg standing (SEO) both before and after the exercises. In Fig 3, the AP velocity in DEC significantly decreased after the exercises (n = 18, p < 0.01, ES = 0.62). The AP velocity significantly differed between DEC and DEO before the exercises (n = 18, p < 0.01, ES = 0.70), but they did not differ between the two conditions after the exercises (n = 18, p = 0.08, ES = 0.28). Both the AP and ML velocities in SEO significantly decreased after the exercises compared with before the exercises (n = 17; AP: p < 0.01, ES = 1.08; ML: p < 0.05, ES = 0.73) as shown in Fig 4.

**Table 1:** Postural sway parameters before and after the exercise.

	Before			After		
	DEO (n = 18)	DEC (n = 18)	SEO (n = 17)	DEO (n = 18)	DEC (n = 18)	SEO (n = 17)
TL (cm)	18.39 ± 6.47	22.01 ± 7.58†	79.26 ± 22.04	18.67 ± 7.36	19.83 ± 5.68	68.94 ± 16.78*
MV (cm/sec)	0.92 ± 0.32	1.10 ± 0.38†	3.96 ± 1.10	0.93 ± 0.37	0.99 ± 0.28	3.45 ± 0.84*
SA (cm^2^)	1.12 ± 1.64	1.15 ± 0.64	6.46 ± 4.12	1.11 ± 0.82	1.00 ± 0.50	5.44 ± 1.93

DEO, double-leg standing with eyes open; DEC, double-leg standing with eyes closed; SEO, single-leg standing with eyes open; TL, total length; MV, mean velocity; SA, sway area.

Values are presented as the means and SDs.

* denotes a significant difference between before and after at p < 0.01.

† denotes a significant difference between DEO and DEC before at p < 0.01. There were significant differences in all of the COP variables between double-leg standing (DEC and DEO) and single-leg standing (SEO) both before and after the exercises.

There were no significant correlations between relative changes in muscle strength after the exercises and relative changes in COP variables of all standing conditions after the exercises (Table 2). For this correlation analysis, the post-exercise values of TFS and PFS that had increased by more than 5% after the exercise than the baseline values were excluded (total 5 subjects).

**Table 2:** Relationships between relative foot and ankle strength after the exercise and relative COP variables while standing after the exercise.

	DEO: correlation coefficients (r)							DEC: correlation coefficients (r)							SEO: correlation coefficients (r)							
	ΔTL	ΔMV	ΔSA	ΔRange		ΔVelocity		ΔTL	ΔMV	ΔSA	ΔRange		ΔVelocity			ΔTL	ΔMV	ΔSA	ΔRange		ΔVelocity	
				AP	ML	AP	ML				AP	ML	AP	ML					AP	ML	AP	ML
ΔTFS (n, 16)	0.14	0.14	-0.15	0.07	-0.25	0.22	-0.13	-0.29	-0.29	-0.06	-0.31	-0.06	-0.48	0.03	ΔTFS (n, 16)	-0.10	-0.10	-0.35	-0.46	-0.05	-0.20	-0.03
ΔPFS (n, 17)	0.17	0.17	0.21	0.15	0.15	0.31	0.01	0.01	0.01	0.15	0.15	0.06	-0.09	0.14	ΔPFS (n, 15)	0.41	0.41	0.02	-0.15	0.13	0.05	0.51

Δ, relative changes after exercises; DEO, double-leg standing with eyes open; DEC, double-leg standing with eyes closed; SEO, single-leg standing with eyes open; TL, total length; MV, mean velocity; SA, sway area; AP, anteroposterior direction; ML, mediolateral direction; TFS, toe flexor strength; PFS, plantar flexor strength. There were no significant correlations between relative changes in muscle strength after the exercises and relative changes in COP variables of all standing condition after the exercises.

## Discussion

This study revealed that fatiguing foot muscle exercises decreased foot muscle strength and altered postural sway during standing. Interestingly, the fatiguing foot muscle exercises decreased the COP range and velocity while standing compared with the pre-fatigue conditions. The decreased foot muscle strength after the exercises was not associated with changed postural sway during standing after the exercises. This result suggests that there is no negative effects of decreased foot muscle strength on maintenance of postural control in double-leg and single-leg standings.

After the exercises, TFS and PFS showed a decrease of 6.9% and 9.3% from before the exercises, respectively. Muscle force production is decreased by impaired neural function, such as decreased motor unit recruitment, especially with the selective reduction of fast-twitch fibers [25]. Selective recruitment of different motor units within synergistic muscles between the gastrocnemius and soleus muscles has been observed during a variety of motor tasks. The gastrocnemius muscle is composed of relatively more fast-twitch fibers, whereas the soleus muscle is composed mainly of slow-twitch fibers [25]. Therefore, the gastrocnemius muscle primarily performs rapid, powerful contractions, whereas the soleus muscle acts in situations involving stability and postural control. In agility and muscle strength exercises, fast-twitch fibers can be activated selectively. Duchateau et al. [26] have shown that myoelectrical activity of the medial gastrocnemius increases to a great extent when the speed of pedaling is increased under a constant load while the myoelectrical activity of the soleus decreases. Conversely, the soleus muscle might be activated during balance ball exercise. Selective activity in the foot and ankle muscles during the exercises would have resulted in relatively small decreases in the maximum muscle strength of the foot and ankle. This small decreased muscle strength might be one of reasons for no impairment of postural sway during standing.

This study showed that fatiguing foot and ankle muscle exercises did not increase in COP variables during double-leg standing and decreased in COP range and velocity during single-leg standing compared with the pre-fatigue conditions. In accordance with our study, other studies have also reported either a decrease or no change in COP variables during standing after the exercises [17,18], although other studies have shown that COP excursion is increased after fatiguing the lower leg muscles [14,15]. These contradictory findings can be explained by the varying effects of different types of exercises on postural sway during upright standing. Also, it has been reported that postural control in double-leg standing requires little muscular activity in the lower limbs [27,28]. This indicates that postural control during double-leg standing after the exercise is regulated by other mechanisms, rather than the muscle force generating capacity. One possible mechanism could be that the fatiguing foot exercises might stimulate the foot sensory input in the postural control system [13]. Postural sway might be improved by increasing the sensitivity of the supportive area of the foot during standing [29]. Additionally, this study showed that visual deprivation (i.e., eyes closed) increased TL and MV as well as the AP velocity during double-leg standing before the exercises; however, these variables did not differ after the exercises. Postural sway became better without vision after the exercise, even though vision is the sensory stimulus that the central nervous system uses most for postural stability [30]. This might support the idea that the exercises improved the somatosensory system of the foot with or without improvement of the vestibular system for postural control. It has been suggested that an increase of reflex activity in the muscle spindles after the exercise might be used for postural control [17]. Furthermore, this study showed no relationships between the relative changes in muscle strength after the exercises and the relative changes in COP variables during standing after the exercises. This suggests that foot muscle strength does not have a major effect on postural control after fatiguing exercises. Based on the local muscle fatigue induced by the exercise, postural sway may not be influenced peripherally; it may have a central origin. Neural effects of the foot and leg muscles after the exercise remain unknown, and further studies should examine these issues to understand the effects of exercise on the neural activity of these muscles.

The direction of the COP trajectory showed different functional characteristics of postural sway during upright standing. Winter [30] has postulated that postural sway in the AP direction is related to ankle joint stability, while postural sway in the ML direction is related to hip joint stability for controlling balance. Based on this strategy, different conditions of the ankle and hip joints could be associated with COP variables in the AP and ML directions. Indeed, postural sway in the AP direction is related to muscle activity at the ankle joint [31], while postural sway in the ML direction may be affected substantially when the ankle stabilizer muscles are fatigued [32]. A previous study shows that a large decrease in the maximum muscle strength of the ankle after the ankle exercises has resulted in an increase of COP velocity in the AP direction but not in the ML direction [33]. On the other hand, our study found a significant decrease in the AP velocity with DEC and in both the AP and ML velocities in SEO after the foot muscle exercises. Another study shows that after performing two types of 30-second core-stability static exercises, COP variables are improved not only in the ML direction but also in the AP direction [34]. The neuromuscular responses after the exercise might help to support both the ankle and hip movements to maintain an upright posture because a coupling of the upper and lower body movements is considered to be regulated by the spinal neuronal networks [35]. Specifically, single-leg standing reduces the base of body support; thus, improvement of postural control in both the AP and ML directions is required to maintain stability [36]. The fatigued foot muscle exercise may modify the postural control system during single-leg standing; a decrease not only in the AP velocity but also in the ML velocity after the fatiguing foot exercises may help to maintain postural control during standing. These results suggest that the fatigued foot muscle exercises may affect the stabilizing subsystem at the ankle and hip (i.e., the control of AP and ML postural sway).

This study has several limitations. Confounding factors that can affect the COP variables, such as different levels of fatigue, muscle activities, and movement control of the joint, were not considered in terms of postural sway while standing. To determine the possible mechanism of postural balance ability after the exercises, further studies should investigate postural sway in relation to activation of the trunk and lower limb muscles while standing. Additionally, it should be considered that fatigue of multiple systems would have a different effect on postural control during standing, i.e., muscle fatigue vs. cardiovascular fatigue. Despite these limitations, our findings help to advance the general understanding of postural control after fatiguing foot exercises. Future studies should investigate the effects of fatiguing muscle exercises on postural control in dynamic situations.

In conclusion, this study demonstrated that fatiguing foot muscle exercises altered postural balance ability; however, decreased foot muscle strength after the exercises was not correlated to altered postural control during standing after the exercises, indicating that decreased foot muscle strength would not possibly have resulted in changed postural control during standing after the exercises and that there would be other forms of postural control involved rather than the foot muscle strength. These results indicate that postural control while standing could be maintained even though foot muscle strength is decreased after fatiguing foot muscle exercises.
